# Small epitope-linker modules for PCR-based C-terminal tagging in *Saccharomyces cerevisiae*

**DOI:** 10.1002/yea.1658

**Published:** 2009-03

**Authors:** Minoru Funakoshi, Mark Hochstrasser

**Affiliations:** Department of Molecular Biophysics and Biochemistry, Yale University266 Whitney Avenue, New Haven, CT 06520-8114, USA

**Keywords:** epitope tagging, PCR, yeast, *Saccharomyces cerevisiae*, pFA6a plasmid, proteasome

## Abstract

PCR-mediated gene modification is a powerful approach to the functional analysis of genes in *Saccharomyces cerevisiae*. One application of this method is epitope-tagging of a gene to analyse the corresponding protein by immunological methods. However, the number of epitope tags available in a convenient format is still low, and interference with protein function by the epitope, particularly if it is large, is not uncommon. To address these limitations and broaden the utility of the method, we constructed a set of convenient template plasmids designed for PCR-based C-terminal tagging with 10 different, relatively short peptide sequences that are recognized by commercially available monoclonal antibodies. The encoded tags are FLAG, 3 × FLAG, T7, His-tag, Strep-tag II, S-tag, Myc, HSV, VSV-G and V5. The same pair of primers can be used to construct tagged alleles of a gene of interest with any of the 10 tags. In addition, a six-glycine linker sequence is inserted upstream of these tags to minimize the influence of the tag on the target protein and maximize its accessibility for antibody binding. Three marker genes, *HIS3MX6, kanMX6* and *hphMX4*, are available for each epitope. We demonstrate the utility of the new tags for both immunoblotting and one-step affinity purification of the regulatory particle of the 26S proteasome. The set of plasmids has been deposited in the non-profit plasmid repository Addgene (http://www.addgene.org).

## Introduction

The model eukaryote *Saccharomyces cerevisiae* has many characteristics that facilitate rigorous genetic and biochemical analyses (Sherman, 2002). One such feature is the high efficiency of homologous DNA recombination. A specific chromosomal region may be replaced efficiently if a DNA fragment bearing homology at its two ends to the target sequence is transformed into yeast cells (Rothstein, 1991). Specific chromosomal sites can also be replaced or tagged by PCR-based methods (Baudin *et al.*, 1993). A common application is the tagging of genes with in-frame sequences encoding epitope tags, which can be recognized by commercially available antibodies, using either one-step (Maeder *et al.*, 2007; Petracek and Longtine, 2002) or two-step gene replacement methods (Moqtaderi and Struhl, 2008; Schneider *et al.*, 1995).

Antibody binding to epitope-tagged proteins can often be improved by adding multiple copies of the epitope. While such large, concatenated epitope tags can enhance antibody affinity or avidity, they also tend to interfere more with the function of the tagged protein (Sabourin *et al.*, 2007). Potential solutions to this problem are the exchange of the tag for another, shorter tag for which higher affinity antibodies are available or the introduction of a flexible linker between the target protein and the tag (Sabourin *et al.*, 2007). Many high-specificity monoclonal antibodies or binding reagents to defined peptide sequences are commercially available (Brizzard, 2008; Waugh, 2005) but relatively few of these are easy to adopt at the moment because of the lack of convenient template plasmids for PCR-based tagging in yeast. It is often desirable to follow several differentially tagged proteins in the same cells, so a broader array of epitope-marker templates for PCR-based epitope tagging would be extremely helpful.

In this paper, we describe a new set of 30 plasmids designed for PCR-based C-terminal tagging with 10 different epitopes using a single set of primers. To minimize interference with the folding and function of the tagged protein, we selected relatively short peptide tags and added a flexible six-glycine linker upstream of the tag. Each tagging vector is available with a *HIS3MX6, kanMX6* or *hphMX4* marker for selection of yeast transformants. We demonstrate the utility of these plasmids by tagging different essential subunits of the yeast 26S proteasome and using the tagged proteins either for immunoblot analysis or affinity purification of proteasomal protein complexes. These plasmids substantially expand the repertoire of peptide epitope tags that can be easily fused to specific proteins in *S. cerevisiae*.

## Materials and methods

### Strains and media

*E. coli* strain TOP10 (Invitrogen) was used for DNA manipulation. Standard bacterial culture media and growth conditions were used (Ausubel, 1987). The yeast strain YPH499 (Sikorski and Hieter, 1989) and its derivatives were used in this study (Table 1), and standard culture media and methods were used for manipulation of yeast cells (Sherman, 2002).

**Table 1:** Yeast strains used in this study

Strain	Genotype	Source
YPH499	*MAT*a *ura3-52 lys2-801 ade2-101 trp1-Δ63 his3-Δ200 leu2-Δ1*	Sikorski and Hieter, 1989
YPH501	*MAT*a*/MAT*α *ura3-52/ura3-52 lys2-801/lys2-801 ade2-101/ade2-101 trp1-Δ63/trp1-Δ63 his3-Δ200/his3-Δ200 leu2-Δ1/leu2-Δ1*	Sikorski and Hieter, 1989
MHY4677	*MAT*a *ura3-52 lys2-801 ade2-101 trp1-Δ63 his3-Δ200 leu2-Δ1 RPT5-6 × GLY-FLAG::kanMX6*	This study
MHY4749	*MAT*a *ura3-52 lys2-801 ade2-101 trp1-Δ63 his3-Δ200 leu2-Δ 1 RPT4-6 × GLY-T7::HIS3MX6*	This study
MHY4913	*MAT*a *ura3-52 lys2-801 ade2-101 trp1-Δ63 his3-Δ200 leu2-Δ1 RPT4-6 × GLY-V5::kanMX6*	This study

### Plasmid constructions

**Figure 1:**
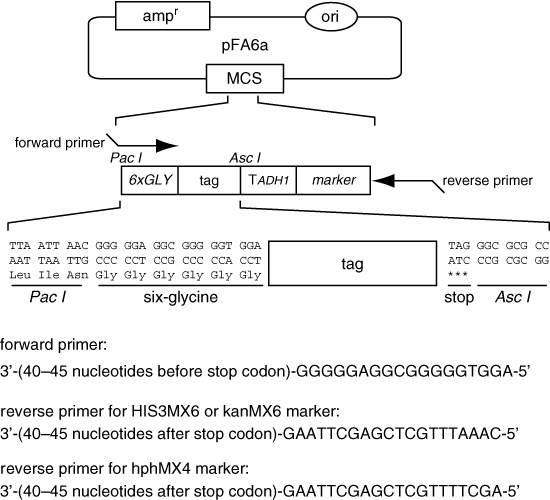
Map of the common template for the series of epitope-tagging plasmids. The positions of the six-glycine coding sequence, epitope tag coding sequence, *ADH1* transcriptional terminator and selection marker between the *Pac*I and *Pme*I restriction sites are shown. The DNA sequences and corresponding translated sequences between *Pac*I and *Asc*I that are common to all the plasmids are also shown. The general design for the forward and reverse primers is shown at the bottom

Conventional methods for plasmid construction were used (Ausubel, 1987). DNA fragments separated in agarose gels were purified by QIAquick gel extraction (Qiagen). Oligonucleotides were synthesized by Integrated DNA Technologies Inc. The plasmids pFA6a–GFP(S65T)–kanMX6 and pFA6a–GFP(S65T)–HIS3MX6 (Wach *et al.*, 1997) were cut with *Pac*I and *Asc*I to remove the GFP(S65T) coding fragment. For construction of all but the longest tag-bearing DNA fragment (3 × FLAG), two complementary oligonucleotides encoding *Pac*I–6 × Gly–tag–*Asc*I were mixed in distilled water. The mixture was heated at 90 °C for 5 min and allowed to cool to 25 °C. The resultant double-strand DNA fragment was mixed with the restricted pFA6a-derived vectors described above and ligated with T4 DNA ligase (New England Biolabs). For construction of the longer *Pac*I–6 × Gly–3 × FLAG–*Asc*I insert, five overlapping primers were annealed: 5′-TAACGGGGGAGGCGGGGGTGGAGACTACA- AAGACCATGACGGTGAT-3′; 5′-TATAAAGAT- CATGACATCGACTACAAGGATGAC-GATGA- CAAGTAGGG-3′; 5′-TTGTAGTCTCCACCCC- CGCCTCCCCCGTTAAT-3′; 5′-GATGTCATGAT- CTTTATAATCACCGTCATGGTCT-3′; and 5′- CGCGCCCTACTTGTC-ATCGTCATCCTTGTA- GTC-3′. These annealed primers were ligated to each other and to the vectors in the presence of T4 DNA ligase and T4 polynucleotide kinase (Fermentas). The sequences of the inserted modules were verified by DNA sequencing. To construct the pFA6a–6 × Gly–tag–hphMX4 plasmids, the 10 pFA6a–6 × Gly–tag–kanMX6 plasmids were cut with *Sac*I and *Bgl*II to remove the *kanMX6* marker gene, and the 1.7 kb *Sac*I–*Bgl*II–*hphMX4* fragment from pAG32 (Goldstein and McCusker, 1999) was inserted. The general structure of the plasmids is shown in Figure 1, and sequences of the tags are given in Table 2.

**Table 2:** Tags and names of plasmids

Tag	Sequence	Plasmid name
FLAG	GAT TAC AAG GAC GAC GAT GAC AAG	pFA6a–6 × GLY–FLAG–HIS3MX6
	Asp Tyr Lys Asp Asp Asp Asp Lys	pFA6a–6 × GLY–FLAG–kanMX6
		pFA6a–6 × GLY–FLAG–hphMX4
3× FLAG	GAC TAC AAA GAC CAT GAC GGT GAT TAT AAA GAT CAT GAC	pFA6a–6 × GLY–3 × FLAG–HIS3MX6
	ATC GAC TAC AAG GAT GAC GAT GAC AAG	pFA6a–6 × GLY–3 × FLAG–kanMX6
	Asp Tyr Lys Asp His Asp Gly Asp Tyr Lys Asp His Asp Ile Asp Tyr Lys	pFA6a–6 × GLY–3 × FLAG–hphMX4
	Asp Asp Asp Asp Lys	pFA6a–6 × GLY–Strep–tagII–HIS3MX6
Strep–tag II	TGG AGC CAC CCG CAG TTC GAA AAA	pFA6a–6 × GLY–Strep–tagII–kanMX6
	Trp Ser His Pro Gln Phe Glu Lys	pFA6a–6 × GLY–Strep–tagII–hphMX4
T7	ATG GCT AGC ATG ACT GGT GGA CAG CAA ATG GGT	pFA6a–6 × GLY–T7–HIS3MX6
	Met Ala Ser Met Thr Gly Gly Gln Gln Met Gly	pFA6a–6 × GLY–T7–kanMX6
		pFA6a–6 × GLY–T7–hphMX4
His tag	CAT CAT CAC CAT CAC CAC	pFA6a–6 × GLY–His–tag–HIS3MX6
	His His His His His His	pFA6a–6 × GLY–His–tag–kanMX6
		pFA6a–6 × GLY–His–tag–hphMX4
S-tag	AAA GAA ACC GCT GCT GCT AAA TTC GAA CGC CAG CAC	pFA6a–6 × GLY–S–tag–HIS3MX6
	ATG GAC AGC	pFA6a–6 × GLY–S–tag–kanMX6
	Lys Glu Thr Ala Ala Ala Lys Phe Glu Arg Gln His Met Asp Ser	pFA6a–6 × GLY–S–tag–hphMX4
Myc	GAG CAG AAA CTC ATC TCA GAA GAG GAT CTG	pFA6a–6 × GLY–Myc–HIS3MX6
	Glu Gln Lys Leu Ile Ser Glu Glu Asp Leu	pFA6a–6 × GLY–Myc–kanMX6
		pFA6a–6 × GLY–Myc–hphMX4
VSV-G	GAG CAG AAA CTC ATC TCA GAA GAG GAT CTG	pFA6a–6 × GLY–VSV–G–HIS3MX6
	Glu Gln Lys Leu Ile Ser Glu Glu Asp Leu	pFA6a–6 × GLY–VSV–G–kanMX6
		pFA6a–6 × GLY–VSV–G–hphMX4
HSV	AGC CAG CCA GAA CTC GCC CCG GAA GAC CCC GAG GAT	pFA6a–6 × GLY–HSV–HIS3MX6
	Ser Gln Pro Glu Leu Ala Pro Glu Asp Pro Glu Asp	pFA6a–6 × GLY–HSV–kanMX6
		pFA6a–6 × GLY–HSV–hphMX4
V5*	AAG CCT ATC CCT AAC CCT CTC CTC GGT CTC GAT TCT ACG	pFA6a–6 × GLY–V5–HIS3MX6
	Lys Pro Ile Pro Asn Pro Leu Leu Gly Leu Asp Ser Thr	pFA6a–6 × GLY–V5–kanMX6
		pFA6a–6 × GLY–V5–hphMX4

* The first Gly of the V5 tag is provided by the last Gly of the 6 × Gly linker.

### PCR amplification of DNA modules and construction of yeast strains

Polymerase chain reactions (PCRs) and yeast transformations were performed as described (Petracek and Longtine, 2002) but with some modifications. Takara EX taq™ (Takara) was used for amplification of DNA fragments from the template plasmids described in Figure 1. The generalized pair of primers used for each PCR is shown in Figure 1, and the specific primers used in the validation studies are given in Table 3. PCR conditions were as follows: a 94 °C, 5 min denaturation step was followed by five cycles of 94 °C, 30 s; 10 °C, 1 min; and 68 °C, 4 min, and then by 30 cycles of 94 °C, 30 s; 65 °C, 30 s; and 72 °C, 4 min. The amplified DNA was used for transformation of diploid yeast strain YPH501 with selection for growth on 0.3 mg/ml Geneticin/G418 (Invitrogen) (for *kanMX6*-marked fragments), 0.3 mg/ml hygromycin B (Roche) (for *hphMX4*-marked fragments), or minimal medium lacking histidine (for *HIS3MX6*-marked fragments). After the recombination was confirmed by colony PCR, the transformants were sporulated and asci were dissected. Single-site insertion of the tagging construct was confirmed by 2 : 2 segregation of the marker gene based on growth on selection plates. Expression of the tagged proteins was verified by immunoblotting.

**Table 3:** PCR primers used in this study

Name	Sequence	Purpose
MF 259	GCTGAAGTTAAGAAATTGGAAGGCACTATAGAATACCA-	*RPT4* C-terminal tagging, forward
	AA AATTAGGGGGAGGCGGGGGTGGA	
MF 232	GTTACTGATATACACATACCTATACATACACATGTCTTT-	*RPT4* C-terminal tagging, reverse
	TTA ACAGAATTCGAGCTCGTTTAAAC	
MF 256	AGTGAAGTTCAAGCAAGAAAATCGAAATCGGTATCCTTTT ATGCAGGGGGAGGCGGGGGTGGA	*RPT5* C-terminal tagging, forward
MF 257	GTAGATATGTGAATGGCGGCTTGATAAATCAAAATATTA-	*RPT5* C-terminal tagging, reverse
	TTA TTTGAATTCGAGCTCGTTTAAAC	
MF 233	ACATAAAAGC TTTGCAAAGT ATTGGACAAT	*RPT4* colony PCR, forward
MF 258	GGTCATGGA TATGAATGAG ATTGAAG	*RPT5* colony PCR, forward
MF 234	AGATCTATATTACCCTGTTATCCCTAGCGG	Colony PCR, reverse

### Immunoblot analysis

Yeast extracts were prepared as described (Kushnirov, 2000). Proteins were resolved by SDS-polyacrylamide gel electrophoresis (SDS–PAGE) and were electrotransferred to Immobilon-P membranes (Millipore). Antibodies against the T7 epitope (1 : 5000, Novagen), V5 epitope (1 : 10 000, Invitrogen), Rpt4 proteasome subunit (1 : 5000, a gift from Dr Thomas Kodadek) Rpn5 proteasome subunit (1 : 5000, a gift from Dr Daniel Finley) and Pre6/α4 20S proteasome subunit (1 : 5000, a gift from Dr Dieter H. Wolf) were used as primary antibodies. Horseradish peroxidase (HRP)-conjugated anti-mouse antibody (GE Healthcare) and HRP-conjugated anti-rabbit antibody (GE Healthcare) were used as secondary antibodies. ECL Western blotting detection reagents (GE Healthcare) were used for protein detection using Kodak Biomax XAR film. Commercial sources for monoclonal antibodies against the other epitope tags described in this report include: FLAG (M2 antibody, Sigma), Strep-tag II (IBA), His tag (anti-tetraHis antibody, Qiagen), S-tag (Novagen), Myc (9E10, Sigma), VSV-G (Sigma), and HSV (Sigma).

### Affinity purification of proteasomal complexes

Yeast cells were frozen in liquid nitrogen and ground to a powder with chilled mortar and pestle as described (Verma *et al.*, 2000; Verma and Deshaies, 2005). A 300 µl aliquot of the powder was thawed in 300 µl buffer A (25 mm Tris–HCl, pH 7.5, 150 mm NaCl, 10% glycerol, 5 mm MgCl_2_, 5 mm ATP). The mixture was centrifuged for 10 min at 21 000 × *g* at 4 °C. The protein concentration of the supernatant was determined using the Bio-Rad protein assay kit, and 700 µg total protein was mixed with 100 µl 50% slurry of FLAG-M2 antibody-agarose beads (Sigma) and incubated for 90 min at 4 °C with constant rotation. The beads were washed three times with buffer A containing 0.2% Triton X-100. The bound proteins were eluted by addition of 50 µl SDS gel sample buffer without DTT. After incubation for 5 min at room temperature, the beads were pelleted and the supernatant was transferred to a new tube. SDS sample buffer containing 0.6 m DTT (25 µl) was added and the samples were heated at 100 °C for 5 min. Aggregated material was pelleted by centrifugation, and the supernatant was used for SDS–PAGE and immunoblotting.

## Results and discussion

### Construction of a set of PCR template plasmids for C-terminal epitope tagging

During our studies of yeast 26S proteasome assembly and function (Kusmierczyk *et al.*, 2008), we found that many of the epitope tags that are available for PCR-based C-terminal epitope tagging (Petracek and Longtine, 2002) compromised the function of this essential proteolytic complex or interfered with its assembly (M.F. and M.H., unpublished data). Many of the commonly used epitope-tagging sequences are fairly long (e.g. 13 consecutive Myc peptides) and they are attached directly to the coding sequence of the target gene. The 26S proteasome is composed of a 20S proteasome core and a 19S regulatory particle bound to one or both ends of the 20S proteasome cylinder, and the 26S complex includes at least 33 different polypeptides (Schmidt *et al.*, 2005). Given the subunit complexity of the proteasome as well as many other protein complexes, it is often useful to tag multiple subunits in the complex in the same cell, and there is still only a limited set of epitope tags that can be employed conveniently for PCR-mediated tagging.

We therefore designed a new series of plasmids for PCR-based C-terminal epitope tag addition in yeast. The following considerations guided our design. First, we chose short peptide epitopes (6–22 residues) that, if possible, had already been shown to have low cross-reactivity with yeast proteins. Shorter tags generally perturb target protein function less than longer ones (Andresen *et al.*, 2004). Second, we introduced a six-glycine (6 × Gly) linker coding sequence upstream of the epitope sequence because previous work had shown that such a flexible oligoglycine linker helps to minimize interference with target protein function (Sabourin *et al.*, 2007). Finally, we used the familiar pFA6a plasmid backbone bearing one of three different markers, *HIS3MX6, kanMX6* or *hphMX4*, which work effectively in the selection for yeast transformants (Petracek and Longtine, 2002; Goldstein and McCusker, 1999).

The *HIS3MX6* and *kanMX6* plasmids were constructed by replacing the GFP(S65T) coding sequence in the pFA6a–GFP(S65T)–HIS3MX6 and pFA6a–GFP(S65T)–kanMX6 plasmids (Wach *et al.*, 1997) with a module encoding the 6 × Gly linker in-frame with one of 10 different peptide epitopes. For the *hphMX4* plasmids, a restriction fragment bearing *hphMX4* was swapped for the *kanMX6* marker fragment in each plasmid of the *kanMX6*-marked set. The resulting plasmids share the structure shown in Figure 1. For subsequent PCR amplification of the tag sequences, the forward primer incorporates the six-glycine coding sequence. The PCR fragments generated are designed to fuse the 6 × Gly-epitope-tag sequence in-frame with the C-terminus of the target protein. The coding sequence for the tag is followed by a stop codon, the *ADH1* transcriptional terminator, and a selectable marker gene. Tag sequences and plasmid names are listed in Table 2.

### Validation of new tagging vectors

**Figure 2:**
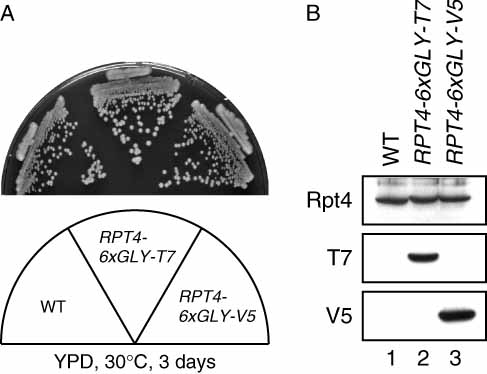
Application of two different epitope tags for detecting the Rpt4 proteasome subunit. (A) Growth of yeast strains expressing the indicated tagged alleles of *RPT4* is indistinguishable from congenic WT cells. The strains used were YPH499 (WT), MHY4749 (*RPT4–6 × GLY–T7*) and MHY4913 (*RPT4–6 × GLY–V5*). (B) Protein extracts from OD_600_ = 0.2 equivalents of the same cells were used for immunoblotting with antibodies against Rpt4, the T7 tag or the V5 tag

To confirm the utility of the new plasmids, we tagged the chromosomal *RPT4* coding sequence with several different 6 × Gly-epitope tags. *RPT4* encodes an essential AAA^+^ ATPase subunit of the 19S regulatory subcomplex within the 26S proteasome (Schmidt *et al.*, 2005). The same two primers were used for all of the amplification reactions (Table 3). Both the *RPT4–6 × GLY–T7* and *RPT4–6 × GLY–V5* derivatives grew at the same rate as the congenic wild-type (WT) strain (Figure 2A). The three strains also grew identically at 37 °C and 16 °C (data not shown). Surprisingly, the *RPT4–6 × GLY–His* yeast strain was non-viable (data not shown), suggesting that some of the tags in Table 2 can interfere with a particular target protein's function. These differences in viability with different tags would have been difficult to predict. This example illustrates why the availability of a range of different epitope tags differing widely in their biochemical properties is advantageous. The expression of the tagged Rpt4 proteins was also examined by immunoblotting (Figure 2B). Based on the anti-Rpt4 blot, the two tagged proteins were expressed at the same level as the untagged Rpt4 subunit, and there was no evidence for proteolytic cleavage of the flexibly linked tags. The epitope-tagged proteins showed strong and specific reactivity with the respective antibodies.

**Figure 3:**
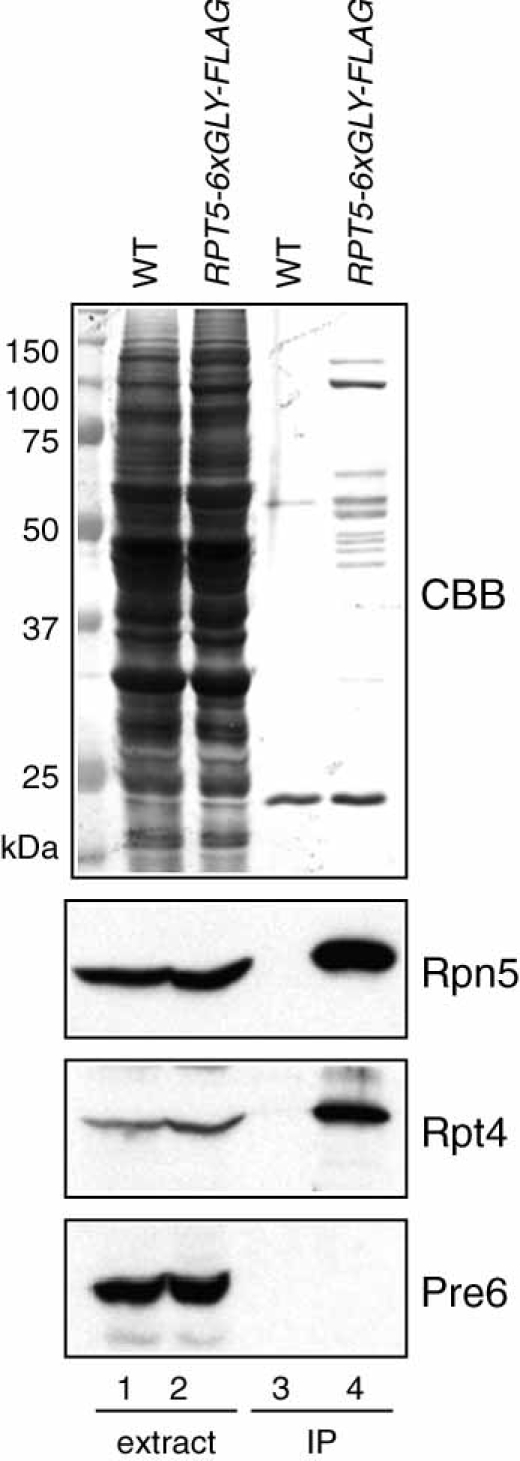
Purification of 19S regulatory particles of the 26S proteasome using a strain expressing the Rpt5–6 × Gly–FLAG protein. Yeast whole cell extracts (10 µg) from YPH499 (lane 1) and MHY4677 (lane 2) and 10 µl proteins eluted from the washed resin (lanes 3 and 4) were used for CBB staining and immunoblotting using antibodies against Rpn5 (lid), Rpt4 (base) or Pre6/α4 (20S core particle). Molecular mass standards are shown at left for the CBB-stained gel

We also tagged another proteasomal ATPase subunit gene, *RPT5*, this time with a sequence encoding a 6 × Gly–FLAG peptide, and determined whether the tagged protein could be used for affinity purification of proteasomal complexes. Whole cell extract from the tagged strain was bound to an anti-FLAG antibody-agarose resin, and the eluate from the resin was analysed by both Coomassie Brilliant Blue (CBB) staining and immunoblotting (Figure 3). Interestingly, a highly purified subset of 26S proteasome subunits could be seen by CBB staining, and based on the pattern of bands, these bands are likely to represent the 19S regulatory complex without the 20S proteasome core. The 19S and 20S proteasomal complexes are usually stably associated when purified in the presence of ATP, which was present in our buffers. The inference that the 19S regulatory complex was specifically isolated was supported by immunoblot analysis. Subunits from the 19S complex, both from the ‘lid’ subcomplex (Rpn5) and the ‘base’ subcomplex (Rpt4) (Schmidt *et al.*, 2005), were detected, but the essential 20S proteasome subunit Pre6/α4 was not (Figure 3, bottom).

Notably, the very C-terminus of Rpt5 bears a conserved motif that is thought to insert into a pocket between specific subunits in the 20S proteasome (Smith *et al.*, 2007), so the C-terminal 6 × Gly–FLAG tag is likely to interfere with this interaction. Cell growth, however, was not impaired (data not shown). FLAG-tagging of other 19S complex subunits led to immunopurification of the entire 26S proteasome (Verma *et al.*, 2000). We conclude that epitope tagging of specific proteasome subunits can facilitate purification of distinct subcomplexes of the proteasome, depending on which subunit is tagged.

## Concluding remarks

In this paper, we have described a new set of plasmids for one-step PCR-based C-terminal tagging of yeast proteins. A single pair of PCR primers can be used for amplification of all 10 tag sequences, which all also include a flexible six-glycine linker. The forward primer in Figure 1 was designed to anneal with the sequence of the six-glycine linker. However, forward primers recommended for previously reported pFA6a-based plasmids (Petracek and Longtine, 2002; Sung and Huh, 2007; Tagwerker *et al.*, 2006; Wach *et al.*, 1997) are also compatible with the new plasmids, although additional missense amino acids will be inserted between the target protein and six-glycine linker. Our plasmids can also be used with a recently described system for C-terminal epitope switching (Sung *et al.*, 2008). Finally, the plasmids marked with the dominant drug resistance genes *kanMX6* and *hphMX4* should be applicable as well for C-terminal tagging in the fission yeast *Schizosaccharomyces pombe* because these selection markers are functional in this organism (Bahler *et al.*, 1998; Marti *et al.*, 2003). The 30 new plasmids, and details about their sequences, are made available at the non-profit plasmid repository Addgene (http://www.addgene.org).
